# Emergence of a novel drinking innovation in an urban population of sulphur-crested cockatoos, *Cacatua galerita*

**DOI:** 10.1098/rsbl.2025.0010

**Published:** 2025-06-04

**Authors:** Barbara C. Klump, David Walter, John M. Martin, Lucy M. Aplin

**Affiliations:** ^1^ Cognitive and Cultural Ecology Research Group, Max Planck Institute of Animal Behavior, Radolfzell am Bodensee, Germany; ^2^ Department of Behavioral and Cognitive Biology, University of Vienna, Vienna, Austria; ^3^ Vienna Cognitive Science Hub, University of Vienna, Vienna, Austria; ^4^ Department of Biology, University of Konstanz, Konstanz, Germany; ^5^ Hawkesbury Institute for the Environment, Western Sydney University, Richmond, New South Wales, Australia; ^6^ Department of Evolutionary Biology and Environmental Studies, University of Zurich, Zurich, Switzerland; ^7^ Division of Ecology and Evolution, Research School of Biology, Australian National University, Canberra, Australian Capital Territory, Australia

**Keywords:** innovation, parrot, urban adaptation, social learning, animal culture, *Cacatua galerita*

## Abstract

The spread of innovation has been proposed as a potentially important source of adaptive behavioural responses to anthropogenic change. Yet, while a diversity of urban innovations have been documented in animals, there are relatively few examples of these spreading to form local traditions. One notable example is the ‘bin-opening innovation’ in sulphur-crested cockatoos (*Cacatua galerita*), where individuals open household bin lids to access food waste, with this behaviour spreading across southern Sydney, Australia. Here, we describe a second innovation in this species, the ‘drinking-fountain innovation’. Individuals from a population in western Sydney drink from twist-handle public drinking fountains, with this behaviour persisting over at least 2 years. Successful operation requires a coordinated sequence of actions, with only 41% of observed attempts ending in success. Intensive observation at one drinking fountain over 44 days revealed 525 attempts and 46% of marked individuals successfully engaging in the behaviour, with individuals visiting at dawn and dusk in line with expectations for use of a water resource. Public drinking fountains vary in design between local councils but are generally widespread. Yet, to our knowledge, this behaviour has not been observed elsewhere. Altogether, this suggests that this drinking innovation has spread to form a new urban-adapted local tradition.

## 1. Introduction

In a rapidly changing world, flexible behavioural responses have been identified as one vital source of adaptive plasticity that allows animals to persist in modified environments [1]. This has been best studied in urban habitats [2], where comparative studies have identified a link between brain size, innovativeness, neophilia and urban adaptation. These findings suggest that, while the trend is not universal, urban habitats generally favour species or individuals that are attracted to novelty and have higher innovativeness [3,4]. For example, studies comparing innovativeness through a proxy of problem-solving between urban and rural populations find a general trend towards better problem-solving in more urban populations [5]. This matches observations of urban innovations ranging from piercing milk bottle tops in great tits (*Parus major*) and blue tits (*Cyanistes caeruleus*) [6], opening sugar packets in Barbados finches (*Loxigilla barbadensis*) [7] and bartering with humans for food in long-tailed macaques (*Macaca fascicularis*) [8].

Yet, innovations will live and die with single individuals unless the novel behavioural patterns are either fixed through natural selection [9] or socially transmitted and culturally inherited [10,11]. The diffusion of innovation through groups and populations to form new cultural traits has therefore been proposed as a potential mechanism by which animals may express rapid, widespread and long-lasting adaptive behavioural change [10,12,13]. Urban habitats may even potentially promote the emergence and spread of innovation, with animals often exposed to more novelty and living in higher social densities [14]. However, empirical examples remain scarce. In a recent review on primates by Gruber *et al.* [12], the authors identified just eight cases of innovations leading to the invention or modification of cultural traits in response to anthropogenic change, five of which involved consuming a new food. Identifying more examples across diverse species and behavioural contexts is therefore paramount to improve our understanding of the factors predicting the emergence of local cultural adaptations to novel environments [10,12].

Despite being globally threatened by habitat loss and the pet trade, parrots are often successful urban adaptors, with invasive and native populations established in cities worldwide [15–17]. Two notable recent studies have described innovative behaviour in urban parrots: first, an introduced population of rosy-faced lovebirds (*Agapornis roseicollis*) in Phoenix, Arizona, uses vents from industrial air conditioning units to cool off and survive the extremely high temperatures of their new urban environment [18]. Second, in a native population of sulphur-crested cockatoos (*Cacatua galerita*, henceforth cockatoos) south of Sydney, cockatoos have innovated how to flip open the household bin lids to access food waste. Subsequent mapping of the social and geographic spread of this behaviour provides clear evidence for the establishment of a local culture [19,20].

**Figure 1.**
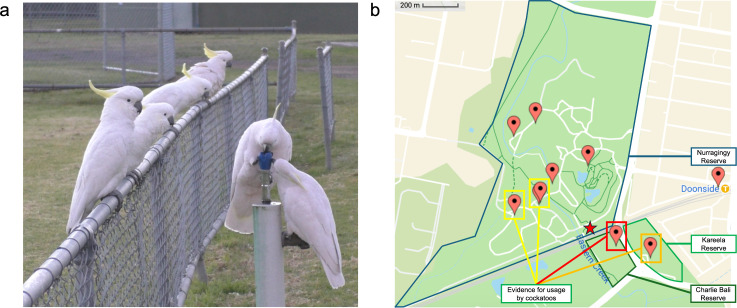
Drinking fountain usage by sulphur-crested cockatoos. (a) Birds interacting with a drinking fountain in Charlie Bali reserve, showing (i) bird operating fountain, (ii) bird scrounging water, and (iii) queuing birds on fence. (b) Map showing the location of 10 drinking fountains of this design identified around the local area. The five highlighted drinking fountains have evidence for usage by cockatoos, with chew marks on the rubber top (yellow, shown in electronic supplementary material, figure S2) and direct observations (orange, red). The camera trap was installed on the drinking fountain in Charlie Ball reserve (red box), nearby to a night roost estimated to be 100−150 individuals (red star).

Here, we describe a second innovation in sulphur-crested cockatoos: accessing water from drinking fountains with a twist-operated handle designed for human use (figure 1a, electronic supplementary material, video S1). We first aim to identify its distribution over space and time, and to examine factors influencing success at the behaviour. We predicted that if the behaviour is still spreading, then this should be reflected in increasing uptake and success over the observation period. We further predicted that consistent with the bin-opening innovation, the behaviour should be male biased [19]. We then explore the potential adaptive benefit of the behaviour, with the starting hypothesis that drinking fountains are reliable water sources when other sources (creeks, puddles, tree-hollows) are dried up. In this case, we predicted that hotter temperatures and drier weather should positively correlate with the expression of the behaviour. Finally, we aim to describe the sequence of actions required to operate drinking fountains, asking whether, similar to Klump *et al.* [19], there is evidence for idiosyncratic individual variation in techniques.

## 2. Methods

### (a) Study site and subjects

This study took place at the neighbouring reserves of Charlie Bali, Kareela and Nurragingy. These reserves are a mix of native vegetation, playground areas and sports fields over approximately 250 ha in western Sydney, Australia. Sulphur-crested cockatoos are a large, highly social parrot common to the wider Sydney area. Local populations share a communal sleeping roost year-round, splitting into smaller foraging flocks in the surrounding area of approximately 2−5 km^2^ [21,22]. At the time of study, there was a night roost of cockatoos at the southern end of the Nurragingy reserve area, estimated to be between 100 and 150 individuals. Data from two GPS-tagged birds over one year suggested that the foraging area for this roost encompassed at least all of Charlie Bali, Kareela and the southern part of Nurragingy reserves, as well as some parts of the nearby suburbs (electronic supplementary material, figure S1).

The drinking-fountain innovation was first observed in September 2018 by B.C.K. during walking transects in western Sydney, and was subsequently also reported to B.C.K. by rangers from a local reserve. In June 2019, we confirmed the ongoing occurrence of the behaviour. We additionally mapped the local distribution of the resource (figure 1b) and located 10 drinking fountains of similar design in the local area, of which seven were in the home range of the local roost (estimated from GPS data, see electronic supplementary material, figure S1).

To be able to visually identify individuals, between 17 July 2019 and 26 September 2019, we habituated and temporarily colour-marked 24 cockatoos close to the roost using a method that does not require capture or restraints (for details see [19,21]). This represented approximately 16–24% of the local population. We additionally caught and GPS-tagged two individuals (TNT, OMG) to gain longer-term information on movements, following methods from [23] (electronic supplementary material, figure S1). Following recommendations of the STRANGE framework [24], we provide demographic details of the colour-marked individuals where known (electronic supplementary material, table S1). Subjects were self-selected as they chose to come close enough to the observer to be marked, potentially meaning our sample of marked birds was biased towards bolder individuals. However, all birds were local residents and had equal possibility to access the drinking fountains.

### (b) Observations and data preparation

Between 29 August and 11 October 2019, we installed two motion-triggered wildlife cameras pointing from either side towards a drinking fountain where we had previously observed cockatoos drinking (figure 1). This drinking fountain, similar to others in the local area, consisted of a rubber top with embedded spout on a concrete stand of approximately 1 m high, with a twist and spring-loaded handle approximately 15 cm from the top. On some fountains, a second twist-handle tap was positioned approximately halfway down the stem (electronic supplementary material, figure S2). The video cameras recorded *ca* 16 000 snippets of 5−15 s in length over the deployment period, of which *ca* 3500 files (*ca* 13.5 h of footage) contained birds. Footage from both cameras was aligned using Movavi Video Editor Plus [25] and compiled into 53 videos of 5−40 min in length for behavioural coding.

### (c) Video scoring and statistical analysis

From video we scored for each attempt by a bird to operate the drinking fountain (with a new attempt starting if the focal bird left the drinking fountain or 60 s passed between video snippets): (i) the time of day for each attempt (to account for changes in daylight hours (+1.5 h) and time of sunrise (−1 h) over the course of data collection, we normalized the time for each attempt compared with the first day of the recording), (ii) whether or not the bird attempting to open was marked and if so, the marking, (iii) the duration of each attempt to the nearest 0.2 s, (iv) whether or not an attempt was successful, (v) whether another bird tried to interfere with the bird attempting to open and (vi) the position of each foot and the beak in relation to the different parts of the drinking fountain (every 0.2 s). For details on video scoring and the ethogram, see electronic supplementary material, supplementary methods and table S2.

#### (i) Distribution over time

The daily number of events per daylight hour (model 1a, to account for change in daylight hours over the data collection period) as well as the daily success rate over time across all birds (model 1b) were assessed using linear regressions (LM, *‘lme4’* package [26]) to investigate whether birds attempted more openings and/or improved in opening the drinking fountain over the observation period.

#### (ii) Factors influencing success

To investigate a potential sex bias, we tested for all birds with known sex, the influence of sex on success (model 2a). For the entire dataset (birds with and without known sex), we ran a generalized linear mixed model (GLMM) with binomial error structure and logit-link function using the *‘lme4’* package [26] to investigate the influence on success of the social environment. We included in the model as fixed effects: (i) the number of birds around, (ii) whether or not another bird had tried to interfere with the focal bird to gain access to the drinking fountain, and (iii) the duration a bird interacted with the drinking fountain, and (iv) as random effect ‘bird ID’ (ID of marked birds or attempt specific number for unmarked birds) to account for data non-independence (model 2b). While both marked and unmarked individuals are represented with multiple opening attempts in our dataset, due to the fact that across time, we only have data on marked birds, we cannot exclude the possibility of hidden replication in the unmarked birds.

#### (iii) Adaptive benefits

To investigate temporal and environmental influences on the number of observed attempts per day, we sourced publicly available data on daily maximum temperature as well as daily rainfall (based on reports from the closest weather station, Horsley Park, Station no. 067119 [27]). Weather data were missing from this station for 10 October 2019; in this case, we used data from Badgerys Creek weather station (Station no. 067108 [27]). We then ran a generalized linear model (GLM) (model 3) using the *‘MASS’* package [28] with a negative binomial error structure. We included as fixed effects: (i) temperature, (ii) rainfall (coded as binary variable with 1 if it rained), and (iv) whether it was a weekday or weekend (to assess the influence of weekly occurrences like sports games).

Model assumptions for all models (1a, 1b, 2a, 2b and 3) were checked using the ‘testResiduals’ function in the package ‘DHARMa’ [29]. Significance of main effects was assessed with likelihood-ratio tests of the best model against the null model at *α* = 0.05.

#### (iv) Individual variation in behavioural action patterns

To test whether individuals showed an idiosyncratic operating technique and whether similarity in outcome (successful or unsuccessful) was associated with a certain operating technique, we split the interaction period of each bird (attempt) into smaller temporal sequences. We defined a sequence as either all interactions of a bird up to the point of a successful opening or the start of a new video snippet (electronic supplementary material, supplementary methods). For further analysis, we subset the dataset to only individuals with at least three operating sequences to ensure repeated opening sequences for all birds in the dataset. We additionally only used sequences with known outcome (*n* = 647 sequences: 440 successful, 207 unsuccessful, by *n* = 89 individuals, of which *n* = 191 sequences were by *n* = 11 colour-marked birds).

**Figure 2.**
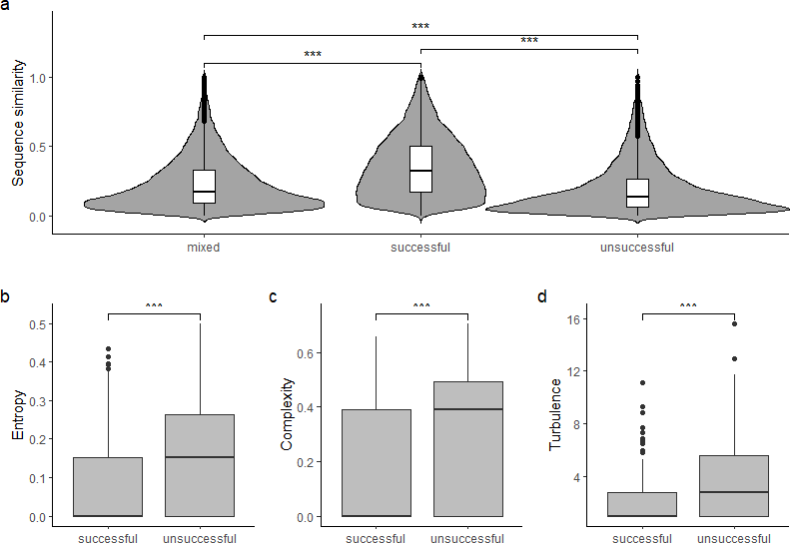
Similarity between sequences based on outcome. (a) Behavioural similarity in sequences between sequences with mixed outcome (one successful and one unsuccessful) or the same outcome (both successful or both unsuccessful). Kruskal–Wallis test: *p* < 0.0001. Comparison of successful versus unsuccessful sequences: (b) within sequence entropy. The Shannon entropy measures the uncertainty of predicting the states in a sequence. If all states are the same, the entropy is zero [33,34]. (c) Complexity index. The complexity index measures the number of transitions in the sequence, reflecting the unpredictability of elements in the sequence [34,35]). (d) Sequence turbulence. Turbulence measures the number of distinct subsequences [34,36].

For each sequence, we converted the behavioural actions scored for left foot, right foot and beak into characters for each 0.2 s, resulting in one string per sequence, encoding both duration and identity of the behavioural actions. We then calculated the Levenshtein distance [30] between all sequences to create a behavioural similarity matrix of all sequences, and created a matching individual and success matrix (binary yes/no). We assessed correlations between the behavioural distance matrix, and the individual and success matrices using the partial Mantel test [31] in the R package ‘vegan’ [32] with the Pearson correlation method. We then compared the behavioural similarity between pairs of sequences with either the same or different outcomes using the Kruskal–Wallis test with *post-hoc* Dunn test. Aiming to understand the composition of the behavioural sequences, we focused only on transitions between actions (ignoring the time component of single actions) to calculate entropy, complexity and turbulence (figure 2) between successful and unsuccessful sequences using the R package ‘TraMineR’ [35].

## 3. Results

Of the 10 drinking fountains in the study area, five exhibited chew marks, giving indirect evidence of usage by cockatoos (figure 1b, electronic supplementary material, figure S2). Direct evidence of operation by cockatoos was gained from camera traps (figure 1a). Overall, these observations showed that individuals operated the drinking fountain using coordinated action with both feet, with one (most often the right) foot on the twist-handle (valve) and one foot gripping the rubber spout (bubbler) or both feet on the valve (electronic supplementary material, figure S2). The weight of the bird would then be lowered to turn the twist-handle clockwise and keep it from springing back and the head turned to access the flowing water.

### (a) Distribution over time

Footage over 44 days recorded 525 operating attempts, of which 105 were by 17 marked birds (mean: 6.2, range: 1−21). Sex was known for 9/17 marked birds (3F, 6M), all of whom operated the fountain (54 attempts: 18F, 36M). Given 24 birds were marked and 17 of them attempted to open the drinking fountain, if marking was unbiased, this suggests that approximately 70% of the local population attempted to operate the drinking fountain. The number of attempts to operate the drinking fountain per day did not significantly change over time (model 1a, LM: *F* = 1.98, *p* = 0.17, *n* = 44). Success rate (percentage of successful openings per day) did not change over the observation period (model 1b, LM: *F* = 0.06, *p* = 0.81, *n* = 44).

### (b) Factors influencing success

Overall, 41% of attempts were successful operations of the drinking fountain. Marked birds showing a slightly higher success rate (52%), but this difference was not significant. There was no influence of sex on success (model 2a, GLMM, *X^2^* = 0.08, df = 1, *p* = 0.78, *n* = 54), but these results should be treated cautiously due to the small sample size.

Direct social interactions (i.e. interference) had no influence on whether an attempt was successful (*p* = 0.35), while longer attempts (*β* = 0.22, *p* = 0.04) as well as attempts where fewer other birds were present (*β* = −0.29, *p* = 0.03) were significantly more likely to be successful (model 2b, GLMM: *X^2^* = 8.49, df = 2, *p* = 0.01, *n* = 525).

### (c) Adaptive value

The usage of the drinking fountain was influenced by time of day, showing a clear bimodal pattern of usage around 07.30 and 17.30, with the highest peak at 17.30 (electronic supplementary material, figure S3). Between days, there was no influence of maximum daily temperature on visitation (*p* = 0.67) and we found no significant difference between weekdays and weekends (*p* = 0.18, note, however, the comparatively small number of weekends in the dataset and a visual trend for fewer attempts during weekends, electronic supplementary material, figure S3). Weather significantly influenced visitation rate, with fewer attempts when it was raining (model 3, *p* < 0.008).

### (d) Individual variation in behavioural action patterns

Successful sequences consisted of in total 39 unique behavioural actions and across the 440 sequences, we observed 119 unique sequences. Across the 207 unsuccessful sequences, we observed 88 unique actions and 131 unique sequences. Successful sequences were on average shorter (electronic supplementary material, table S3). Interestingly, the two most common sequences in both outcomes (without repetitions) were identical (electronic supplementary material, table S4).

The operating technique of individuals was idiosyncratic in that sequences by one individual were more similar than sequences by different individuals (partial Mantel test, accounting for success: *r* = 0.22, *p* < 0.001). Sequences with the same outcome were more similar than sequences with different outcomes (successful–unsuccessful) even when accounting for individual identity (partial Mantel test: *r* = 0.07, *p* < 0.001). *Post*-*hoc* analysis showed that sequences with successful outcomes were more similar to each other than unsuccessful sequences were to each other or sequences with mixed outcomes (figure 2a, Dunn test, *p*-values adjusted for multiple testing, all *p* < 0.0001). Accounting for individual variation, successful sequences showed significantly less variation in composition than unsuccessful sequences in entropy (LMM: *X^2^* = 21.15, df = 1, *p* < 0.0001, *n* = 647, figure 2b), the number of transitions (complexity, LMM: *X^2^* = 17.10, df = 1, *p* < 0.0001, *n* = 647, figure 2c) and sequence turbulence (LMM: *X^2^* = 26.35, df = 1, *p* < 0.0001, *n* = 647, figure 2d).

## 4. Discussion

Here, we identify a drinking innovation in wild parrots, the first such to our knowledge. The behaviour consists of a combination of actions involving both feet, bill and shifting body weight to start the water flow. This apparent complexity in behaviour is potentially reflected in our finding that while it appeared to be well-established in the local population, only 52% of attempts by marked birds to operate the drinking fountain were successful. This has interesting parallels to an earlier study on bin-opening in cockatoos where 54% of attempts by marked birds were successful [19], suggesting similarities in either their physical difficulty or time taken to learn. However, unlike the bin-opening innovation, where 32% of marked individuals in the local population attempted, here an estimated 70% of marked individuals attempted, with no evidence for ongoing spread. This suggests that the drinking fountain innovation had already undergone extensive social diffusion prior to the study. In contrast with Klump *et al.* [19], where the bin-opening was heavily biased towards males, we observed no sex bias in attempts to use, or success at, the drinking fountain. This might suggest that innovativeness *per se* does not vary between sexes, but rather is the result of an extrinsic difference between the resources. For example, bin-lids might require more physical strength to open than drinking fountains, modifying the cost–reward trade-off for smaller females. Alternatively, competition for bins may be higher, biasing opening towards dominant males [37]. In support of this second hypothesis, while we observed extensive queuing for drinking fountains, water at drinking fountains (unlike waste in bins), is effectively infinite. Thus, while we observed higher success rates when fewer birds were around, all individuals could feasibly eventually access water.

More similarly with the previously described bin-opening behaviour [19], the drinking-fountain innovation shows a strong individual signature, with individuals performing combined actions to (attempt to) open the valve in an idiosyncratic way. This suggests that individual learning at least partly plays a role in the acquisition of the behaviour. However, when accounting for these idiosyncratic differences, it became clear that opening techniques that were successful showed significantly less variability in various measures of composition (figure 2). While we can currently only speculate about what underlies this variation, the exploratory nature of cockatoos [38,39] makes it conceivable that individuals who have not mastered the opening are trying more different options, leading to the observed patterns.

Foraging innovations have been identified across multiple animal taxa [40–42], and the spread of foraging innovations to form local traditions has been described in several bird and mammal species [19,43–45]. Yet, drinking innovations appear to be rare. Perhaps the best example comes from chimpanzees where individuals began utilizing ‘moss-sponges’ to scoop water, with this behaviour spreading through the group [46]. In this case, moss-sponges replaced habitual ‘leaf-sponges’ and were suggested to be advantageous in a higher competitive environment. In our case, it is less clear why the drinking-fountain behaviour has been adopted. We initially hypothesized that the adaptive advantage of drinking fountains might be that they represent a more reliable water source than puddles, creeks or tree-hollows, which might dry-up under hot conditions. However, use of the drinking fountain was regular, extensive and not restricted to hotter days, as expected if it was a supplement to less reliable water sources [47,48]. Alternative hypotheses could include that drinking-fountain water tastes better than alternatives [49], that its use represents contrafreeloading behaviour [50], or that the placement of drinking fountains in open areas provides anti-predator benefits [51]. These remain to be tested.

Despite this habitual use, there was no evidence of usage of drinking fountains outside the five contained within the home range of this single roost. For instance, while we did not specifically survey other areas, we did not receive any reports from the public (i.e. through the citizen science app, *BigCityBirds,* supplementary text). This is in dramatic contrast to Klump *et al.* [19], where bin-opening had spread from three to 44 suburbs in several years and is still widely reported by people on *BigCityBirds*. However, further consideration of the resource may provide the answer. Wheeled household bins are almost ubiquitous across Australia, with bins varying little in design. In contrast, while public drinking fountains are commonly provided in residential areas, their design tends to be locally standardized but variable between different local councils. Unfortunately, data on the surrounding local council areas are unavailable, but for example, the additional public fountains provided by *Sydney Water* and distributed across the wider Sydney area consist of a horizontal tray with push button, requiring a very different set of behavioural actions to access. It therefore seems most likely that the wider resource landscape, rather than social connectivity, cognitive limitations or learning biases, is the factor constraining the geographic spread of this innovation.

In summary, we describe a new innovation in urban-living cockatoos, the ‘drinking-fountain innovation’. This behaviour appears to be widely adopted in the local population, suggesting it has spread through social learning to establish as a local tradition. A long history of comparative and captive studies has shown that parrots are capable of solving tasks requiring complex motor actions [52]. Yet, their cognitive ecology remains understudied in the wild. Our study adds to two recent descriptions of innovations in urban-living parrots; opening bins to access human food waste [19] and using vents from industrial air conditioning units to survive high urban temperatures [18]. Together, these examples evidence how the spread of innovation may be a key mechanism in some parrot species for adaptive behavioural responses to the variable challenges and opportunities of anthropogenic changes.

## Data Availability

Data and code are available at Edmond, the open access repository of the Max Planck Society [53]. Supplementary material available online [54].
